# Recurring incursions and dissemination of novel Eurasian-origin H5Nx avian influenza viruses in Atlantic Canada

**DOI:** 10.1093/ve/veae111

**Published:** 2024-12-17

**Authors:** Ishraq Rahman, Cassidy N.G Erdelyan, Anthony V Signore, Ifeoluwa Ayilara, Jordan Wight, Megan E.B Jones, Daniel S Sullivan, Oliver Lung, Tamiko Hisanaga, Sabina I Wilhelm, Joshua T Cunningham, Christopher R.E Ward, Johanna Bosch, Gregory J Robertson, Karen Gosse, Meghan Baker, Beverly Dawe, Stéphane Lair, Jennifer F Provencher, Kathryn E Hargan, Yohannes Berhane, Andrew S Lang

**Affiliations:** Department of Biology, Memorial University of Newfoundland, St. John’s, NL A1C 5S7, Canada; National Centre for Foreign Animal Disease, Canadian Food Inspection Agency, Winnipeg, MB R3E 3M4, Canada; National Centre for Foreign Animal Disease, Canadian Food Inspection Agency, Winnipeg, MB R3E 3M4, Canada; National Centre for Foreign Animal Disease, Canadian Food Inspection Agency, Winnipeg, MB R3E 3M4, Canada; Department of Biology, Memorial University of Newfoundland, St. John’s, NL A1C 5S7, Canada; Canadian Wildlife Health Cooperative, Atlantic Region, Charlottetown, PEI C1A 4P3, Canada; Atlantic Veterinary College, University of Prince Edward Island, Charlottetown, PEI C1A 4P3, Canada; National Centre for Foreign Animal Disease, Canadian Food Inspection Agency, Winnipeg, MB R3E 3M4, Canada; Department of Biological Sciences, University of Manitoba, Winnipeg, MB R3T 2M5, Canada; National Centre for Foreign Animal Disease, Canadian Food Inspection Agency, Winnipeg, MB R3E 3M4, Canada; Department of Biological Sciences, University of Manitoba, Winnipeg, MB R3T 2M5, Canada; National Centre for Foreign Animal Disease, Canadian Food Inspection Agency, Winnipeg, MB R3E 3M4, Canada; Canadian Wildlife Service, Environment and Climate Change Canada, Mount Pearl, NL A1N 4T3, Canada; Wildlife Research Division, Environment and Climate Change Canada, Mount Pearl, NL A1N 4T3, Canada; Canadian Wildlife Service, Environment and Climate Change Canada, Mount Pearl, NL A1N 4T3, Canada; Wildlife Research Division, Environment and Climate Change Canada, Mount Pearl, NL A1N 4T3, Canada; Wildlife Research Division, Environment and Climate Change Canada, Mount Pearl, NL A1N 4T3, Canada; The Rock Wildlife Rehabilitation Centre, Torbay, NL A1K 1A5, Canada; Animal Health Division, Department of Fisheries, Forestry and Agriculture, Government of Newfoundland and Labrador, St. John’s, NL A1E 3Y5, Canada; Animal Health Division, Department of Fisheries, Forestry and Agriculture, Government of Newfoundland and Labrador, St. John’s, NL A1E 3Y5, Canada; Centre Québécois sur la Santé des Animaux Sauvages/Canadian Wildlife Health Cooperative, Faculté de Médecine Vétérinaire, Université de Montréal, St. Hyacinthe, QC J2S 2M2, Canada; Environment and Climate Change Canada, Science and Technology Branch, National Wildlife Research Centre, Carleton University, Ottawa, ON K1A 0H3, Canada; Department of Biology, Memorial University of Newfoundland, St. John’s, NL A1C 5S7, Canada; National Centre for Foreign Animal Disease, Canadian Food Inspection Agency, Winnipeg, MB R3E 3M4, Canada; Department of Veterinary Pathology, Western College of Veterinary Medicine, University of Saskatchewan, Saskatoon, SK S7N 5B4, Canada; Department of Animal Science, University of Manitoba, Winnipeg, MB R3T 2N2, Canada; Department of Pathobiology, Ontario Veterinary College, University of Guelph, Guelph, ON NIG 2W1, Canada; Department of Biology, Memorial University of Newfoundland, St. John’s, NL A1C 5S7, Canada

**Keywords:** gulls, viral dissemination, clade 2.3.4.4b, H5N5, highly pathogenic avian influenza

## Abstract

Wild birds are important hosts of influenza A viruses (IAVs) and play an important role in their ecology. The emergence of the A/goose/Guangdong/1/1996 H5N1 (Gs/GD) lineage marked a shift in IAV ecology, leading to recurrent outbreaks and mortality in wild birds from 2002 onwards. This lineage has evolved and diversified over time, with a recent important derivative being the 2.3.4.4b sub-lineage, which has caused significant mortality events in wild bird populations. An H5N1 clade 2.3.4.4b virus was transmitted into North America from Eurasia in 2021, with the first detection being in Newfoundland and Labrador in Atlantic Canada, and this virus and its reassortants then spread broadly throughout North America and beyond. Following the first 2021 detection, there have been three additional known incursions of Eurasian-origin strains into Atlantic Canada, a second H5N1 strain in 2022 and two H5N5 strains in 2023. In this study, we document a fifth incursion in Atlantic Canada that occurred in 2023 by another H5N5 strain. This strain spread throughout Atlantic Canada and into Quebec, infecting numerous species of wild birds and mammals. Genomic analysis revealed mammalian-adaptive mutations in some of the detected viruses (PB2-E627K and PB2-D701N) and mutations in the hemagglutinin (HA) and neuraminidase (NA) genes that are associated with enhanced viral fitness and avian transmission capabilities. Our findings indicate that this virus is continuing to circulate in wildlife, and confirms Atlantic Canada is an important North American entry point for Eurasian IAVs. Continued surveillance and genomic analysis of IAVs detected in the region is crucial to monitor the evolution of these viruses and assess potential risks to wildlife and public health.

## Introduction

Wild birds are a key reservoir for influenza A viruses (IAVs), hosting most of the known IAV genetic diversity. For example, considering only the hemagglutinin (HA)-encoding gene, 17 of the 19 known subtypes circulate among wild birds (Olsen et al. 2006, Fereidouni et al. 2023), while the remaining two have only been identified in bats (Wu et al. 2014). Wild birds usually appear asymptomatic when infected by IAVs, with these viruses classified as low pathogenic avian influenza viruses (LPAIVs) based on their pathogenicity in chickens (Swayne and Suarez 2000). Highly pathogenic avian influenza viruses (HPAIVs) can evolve from LPAIVs belonging to HA subtypes H5 and H7 through the evolution of a polybasic cleavage site in the HA gene, which leads to broader tissue tropism and increased virulence (Swayne 2007, Stech and Mettenleiter 2013, de Bruin et al. 2022). The emergence of the A/goose/Guangdong/1/1996 H5N1 (Gs/GD) lineage of HPAIV in 1996 (Guo et al. 1998) was a globally significant event, as it led to recurring outbreaks in wild birds from 2002 onwards (Ramey et al. 2022, Xie et al. 2023). This lineage has diversified over time through continued evolution of new sub-lineages of the H5 gene and reassortments (Xie et al. 2023). Particularly important in relation to wild birds was the evolution of a new sub-lineage 2.3.4.4 (Smith et al. 2015) and its sub-clade 2.3.4.4b (The Global Consortium for H5N8 and Related Influenza Viruses 2016), which first appeared in 2016 and subsequently has spread globally among wild birds (Xie et al. 2023).

In 2020, the landscape for HPAIV in wild birds shifted once more when viral circulation persisted in wild birds in Europe through the entire year, whereas it had previously caused repeated but finite outbreaks in this region (Lewis et al. 2021, Pohlmann et al. 2022, Xie et al. 2023, Bøe et al. 2024). This was followed by transmission of H5N1 HPAIVs from Eurasia into North America via both the Atlantic (Caliendo et al. 2022) and Pacific (Alkie et al. 2022) routes. The Atlantic introduction was first detected on the Avalon Peninsula of Newfoundland and Labrador, the easternmost point of the continent, in late 2021. This virus and its derivatives then rapidly spread throughout the America, and eventually to Antarctica (Kandeil et al. 2023, Leguia et al. 2023, Ruiz-Saenz et al. 2023, Youk et al. 2023, Giacinti et al. 2024). Subsequently, additional incursions of new H5N1 strains of Eurasian-origin were detected again on both the Atlantic and Pacific sides of North America in 2022 (Alkie et al. 2023a, Ramey et al. 2024), and then more recently two novel H5N5 strains were detected in Atlantic Canada in 2023 (Erdelyan et al. 2024).

The arrivals of these viruses have resulted in unusual mass-mortality events in multiple species and locations in the America, notably in colonial nesting seabirds such as Northern Gannets (*Morus bassanus*) and Peruvian Pelicans (*Pelecanus thagus*) (Leguia et al. 2023, Avery-Gomm et al. 2024, Lane et al. 2024). There have also been repeated transmissions of these viruses from birds to mammals, particularly to small carnivores (Chestakova et al. 2023, Alkie et al. 2023b, Erdelyan et al. 2024), which presumably were infected via scavenging, as well as numerous species of marine mammals (Leguia et al. 2023, Ulloa et al. 2023, Lair et al. 2024, Plaza et al. 2024).

Surveillance in live and dead wild birds has been ongoing in Atlantic Canada since the initial detection of H5N1 in late 2021 (Giacinti et al. 2024), and through these efforts we have detected an additional, fifth incursion from Eurasia by another distinct H5N5 strain. This strain was once again first detected on the Avalon Peninsula in Newfoundland and Labrador but has since spread among wild birds throughout Atlantic Canada, to Quebec, and been transmitted to mammals. The goal of this study is to document this fifth incursion, describe its dissemination, and present the important mutations detected in this strain through genomic analysis.

## Materials and methods

### Ethics

Work at Memorial University was conducted under biosafety permit S-103 from its Institutional Biosafety Committee. All samples used in the study were collected from dead animals.

### Sampling and initial screening for influenza A virus

Oropharyngeal and cloacal swabs were taken from bird carcasses and placed in Multitrans viral transport media (Starplex Scientific). Brain tissue samples were also taken from most necropsied birds from Newfoundland and Labrador. Oral swabs were taken from mammals. Sample processing and IAV screening were performed as previously described (Erdelyan et al. 2024, Wight et al. 2024). All IAV matrix-positive samples were submitted to the National Centre for Foreign Animal Disease (NCFAD) laboratory in Winnipeg for confirmatory testing.

### Viral isolation, genomic sequencing, and assembly

For virus isolation, IAV PCR-positive samples were propagated through the allantoic cavities of 9-day-old embryonated-specific pathogen-free (SPF) chicken eggs (Terregino and Capua 2009). The samples used for genomic sequencing, including passage history where appropriate, are detailed in Supplementary Table S1. Total RNA was extracted from samples using the MagMAX-96 Viral RNA Isolation Kit with the KingFisher Duo Prime platform (ThermoFisher Scientific). The presence of IAV genomic material was verified using the matrix gene-specific RT–qPCR (Weingartl et al. 2010), followed by modified H5-specific RT–qPCR (Spackman et al. 2002). The eight genomic segments were amplified as described previously (Zhou et al. 2009), and sequencing and assembly were also performed following previously described methods (Alkie et al. 2022). Briefly, amplicons were subjected to library preparation using the Rapid Barcoding Kit (Oxford Nanopore; SQK-RBK110.96) and sequenced using MinION R9.4.1 Flow Cells on an Oxford Nanopore GridION sequencer (Oxford Nanopore Technologies). Samples processed since 17 January 2024 have been sequenced with the Rapid Barcoding Kit (Oxford Nanopore; SQK-RBK114.96) and MinION R10.4.1 Flow Cells. Raw sequence data were basecalled and demultiplexed with Guppy (v6.4.6) using the super accurate model. In December 2023, the basecaller was switched from Guppy to Dorado (v7.2.12). Basecalled reads were processed and analysed with the CFIA-NCFAD/nf-flu pipeline (v3.3) (Kruczkiewicz et al. 2024) against all sequences from the National Center for Biotechnology Information (NCBI) Influenza Virus Sequence Database and selected influenza virus sequences from the Global Initiative on Sharing All Influenza Data (GISAID) and internally generated sequences from the ongoing 2021–24 H5Nx outbreak with a 10× cutoff. Geneious Prime 2023.0.4 with Minimap2 (v2.2.4) were used for additional analysis (Li and Birol 2018). Sequences containing >90% of the coding regions were retained for further phylogenetic analysis. The 20 genomic sequences have been deposited on GISAID under Isolate IDs: EPI_ISL_19260099 to EPI_ISL_19260118. The sequences have also been submitted to NCBI SRA (BioProject ID PRJNA1182792).

### Sequence collection and analyses

Additional sequences were retrieved from the NCBI Influenza Virus Sequence Database and the GISAID EpiFlu database (Shu and McCauley 2017). Sequences from GISAID are listed in Supplementary Table S2. H5N5 virus sequences similar to A/swan/Rostov/2299-2/2020 (available as of 30 April 2024) and possessing eight segments were examined and reassortants were removed (*n* = 8; no NA stalk deletion) resulting in 119 whole-genome sequences (WGS). Accession numbers are listed in Supplementary Table S2.

Sequences were aligned using MAFFT v7.490 and trimmed of non-coding sequences. The 119 WGSs were concatenated and assessed for reassortment using Recombination Detection Program (RDP) (v5.58 Beta) (Martin et al. 2021) using the default settings except for selection of linear sequences. The sequences were again checked using all detection methods (RDP, GENECONV, Chimaera, MaxChi, BootScan, SiScan, 3Seq, LARD) screening for any regions detected by >1 method. Maximum-likelihood trees and best-fitting nucleotide substitution models, identified using ModelFinder (Kalyaanamoorthy et al. 2017), were inferred using IQ-TREE v2.3.6 (Nguyen et al. 2015) and 1000 replicates were used for the Shimodaira–Hasegawa approximate likelihood ratio test. For the WGS, IQ-TREE was run with concatenated sequences, and a partition file for the individual DNA segment locations. TreeTime (v0.11.4) (Sagulenko et al. 2018) was used to reconstruct the most likely ancestral sequence for HA and the WGS, specifically the codon for the mature H5-156 residue.

The H5N5 WGS were used to estimate the timing and likelihood of transmissions between geographic locations in discrete space using BEAST v1.10.4 (Lemey et al. 2009, Suchard et al. 2018). The WGS alignments were used to estimate a time-scaled phylogenetic tree under the GTR + I + Γ4 nucleotide substitution model (Tavaré and Miura 1986), a relaxed molecular clock with lognormal distribution, and an exponential population coalescent tree prior. Sampling locations [the Canadian provinces of New Brunswick (NB), Nova Scotia (NS), Prince Edward Island (PEI), and Quebec (QC), and the countries Norway, Bulgaria, Romania, Russia, Iceland, England, Scotland, the Netherlands, Germany, and Japan (Hokkaido and Iwate)] were attached to each tree tip as a discrete character state in BEAST v1.10.4 (Lemey et al. 2009, Suchard et al. 2018).

Ancestral character states for location were reconstructed by the asymmetric substitution model and social networks were inferred with the Bayesian stochastic search variable selection (BSSVS) procedure. Four independent Markov Chain Monte Carlo chains (200,000,000 steps, sampled every 20,000) were run. The first 10% of samples from each chain were burned and assessed for convergence (effective sample size >200) using Tracer v1.7.2. Post burn-in samples from independent chains were combined using LogCombiner v1.10.4 and a maximum clade credibility (MCC) tree was produced using TreeAnnotator v1.10.4 (Suchard et al. 2018). The MCC tree has branch posterior probabilities ≥0.60 annotated. The posterior distribution of indicator values from the BSSVS procedure was used to conduct Bayes factor (BF) tests to garner statistical support for location transitions using SpreaD3 (v0.9.7.1) (Bielejec et al. 2016). Transitions with BF <3.0 were discarded from the dataset. The MCC tree was used to plot location state transitions on a world map with SpreaD3. The width of each transition line was manually adjusted to represent the underlying BF support (substantial support: 3.0 ≤ BF < 10.0, very strong support: 10.0 ≤ BF < 100.0, and decisive support: BF ≥ 100.0) (Lemey et al. 2009, Hicks et al. 2019). The above Bayesian methods were repeated on a reduced sequence dataset, with a maximum of three sequences per regional monophyletic clade (Supplementary Fig. S1).

All resultant phylogenetic trees were visualized using ggtree (Yu et al. 2017).

## Results and discussion

### Fifth incursion and dissemination of a Eurasian-origin 2.3.4.4b clade H5 virus in Atlantic Canada

A dead Herring Gull (*Larus argentatus*) was found on Gull Island in the Witless Bay Ecological Reserve, Newfoundland and Labrador, in June of 2023 that was infected by an H5N5 strain. This was the only AIV-positive sample from Newfoundland and Labrador from June to early September of 2023. Between 28 September and 10 November 2023, five Herring Gulls, one Ring-billed Gull (*Larus delawarensis*), and five Great Black-backed Gulls (*Larus marinus*) tested positive for H5N5 in the region. While most were found near St. John’s, Newfoundland, one individual was found 28 September 2023 on an offshore oil and gas production platform ∼350 km east of St. John’s, suggesting widespread circulation among gulls in the region. On 20 November 2023, thre dead Great Black-backed Gulls were found washed ashore near Port Caledonia, Nova Scotia and were infected with the same strain. The virus clearly continued to circulate in the Atlantic region for the next several months and was also identified in Striped Skunks (*Mephitis mephitis*), Raccoons (*Procyon lotor*), and American Crows (*Corvus brachyrhynchos*) found dead through April 2024. The virus also disseminated across other eastern Canadian provinces, with detections in wildlife found dead in Prince Edward Island and Quebec.

Genome-wide comparison of recent and past H5N5 sequences from Atlantic Canada and viruses in the GISAID database show that this virus first detected in Newfoundland in June 2023 was another novel incursion, and not continued spread and dissemination of the earlier H5N5 viruses (Fig. 1 and Supplementary Fig. S1). No recombination in the concatenated WGS was detected by RDP. All individual segment ML trees displayed similar topology, sharing a most recent common ancestor with 2022 Norwegian sequences (Supplementary Fig. S2). The recent Canadian sequences clustered distinctly from those previously reported, indicating a novel incursion. Sequences from this incursion were more similar to viruses identified in the northeastern Atlantic Ocean (Iceland, England, Scotland, the Netherlands, Germany, and Norway) and the northwestern Pacific Ocean (Japan) regions through late 2023 and early 2024, than to those previously detected in Canada. Therefore, this represents the fifth incursion of a novel 2.3.4.4b clade virus from Eurasia into Atlantic Canada.

**Figure 1.**
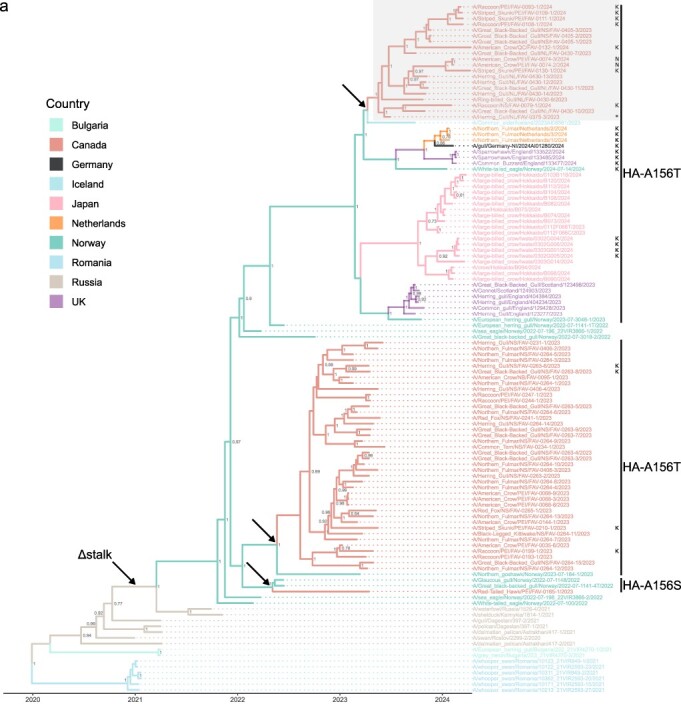
Bayesian time-resolved MCC tree of H5N5 WGS. (a) H5N5 viruses similar to the first detection, A/swan/Rostov/2299-2/2020 (H5N5). The origin of the Δstalk genotype is indicated. Arrows indicate incursions into Canada. K and N denote PB2-E627K and PB2-D701N mutations, respectively. Mature H5 numbering is used for HA residues. The shaded region identifies the new sequences generated in this study. (b) Zoom-in on the newly generated sequences from the most recent incursion of H5N5-Δstalk viruses detected in eastern Canada. The first detection in Newfoundland is marked with an asterisk (*). Values are posterior probabilities. Canadian detections are indicated by province (NL, Newfoundland and Labrador; NS, Nova Scotia; PEI, Prince Edward Island; QC, Quebec; NB, New Brunswick).

**Figure 1.**
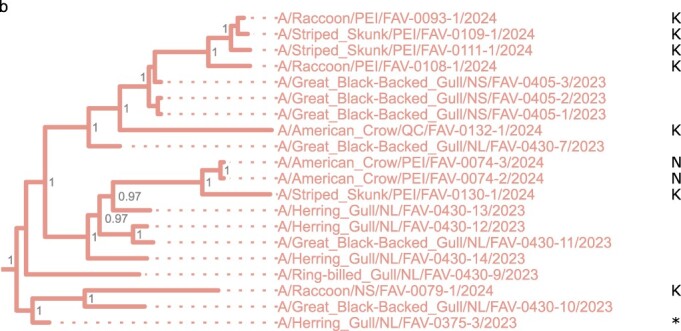
Continued

As observed with the previous H5N1 and H5N5 incursions (Alkie et al. 2023a, Erdelyan et al. 2024, Giacinti et al. 2024, Signore et al. 2024), this H5N5 virus infected multiple species across multiple provinces in eastern Canada. Given that at the time of the preparation of this manuscript this strain was detected as recently as April 2024, this suggests that the virus is still circulating in wildlife along with other AIVs. Thus, there is the potential for this virus to undergo reassortment with other circulating AIVs, as has been documented for H5N1 (Kandeil et al. 2023, Signore et al. 2024).

### Timeline of H5Nx virus incursions into Atlantic Canada

**Figure 2.**
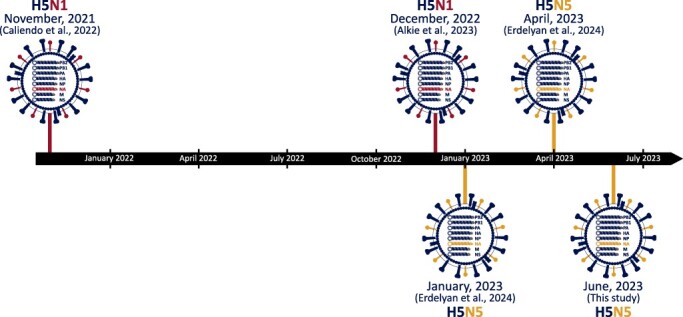
Timeline of Eurasian H5Nx virus incursions into Atlantic Canada.

The five incursions of 2.3.4.4b clade viruses from Eurasia into Atlantic Canada are depicted in Fig. 2. The first H5N1 incursion in November 2021 (Caliendo et al. 2022) was followed by detection of a second H5N1 incursion a year later, in December 2022 (Alkie et al. 2023a). The H5N1 viruses from the second incursion were similar to European H5N1 viruses belonging to the B2 HA sub-lineage, while the initial H5N1 viruses belonged to the B1 HA sub-lineage (Pohlmann et al. 2022, Alkie et al. 2023a). A few months later in January 2023, the first detection of an H5N5 virus in Atlantic Canada was in an American Crow (Erdelyan et al. 2024), and related viruses continued to be detected in several bird and mammal species in the following months. In April 2023, a genetically distinct H5N5 was detected in a Red-tailed Hawk (*Buteo jamaicensis*), representing a second incursion of a novel H5N5 virus into Atlantic Canada (Erdelyan et al. 2024), although this was the only detection of this second H5N5 virus. The H5N5 strain detected in the Herring Gull found in the Witless Bay Ecological Reserve in Newfoundland and Labrador in June 2023 represents the latest, and the fifth, incursion of a novel 2.3.4.4b clade virus into Atlantic Canada (Figs 1 and 2). Taken together, these incursions, which have now been shown to be separate events separated by months in some cases, and not spillovers from a single event, demonstrate that there are recurring transmission events via migratory birds to eastern Canada for viruses originating in European wildlife.

### Host and geographic distributions of the H5N5 viruses

**Figure 3.**
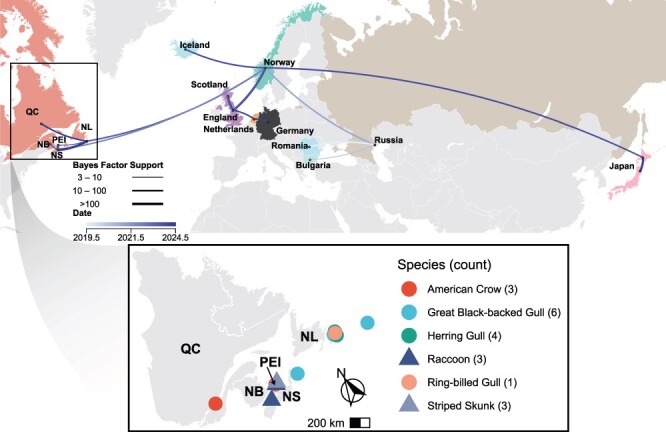
Phylogeographic reconstruction of H5N5 dispersal since the first detection in Russia in 2020 and detection of H5N5 infections during the most recent incursion into eastern Canada by location and species (numbers of infected individuals for each host is in brackets). The introductions into Atlantic Canada in 2023 are most closely associated with viruses that were circulating in Norway. The curve of the line indicates direction of dispersal in a clockwise direction. Locations are generally mapped in the centre of the province/country for visualization purposes. The earlier Canadian introduction was two discrete viruses (January and April 2023; Figs 1 and 2), one of which was only detected once.

Considering the three independent H5N5 incursion events, these viruses have disseminated broadly in terms of infected species (Figs 1 and 3). There have been 61 detections of H5N5 in Canada as of April 2024, spanning all Canadian provinces that have coastlines on the Atlantic Ocean/Gulf of St. Lawrence, 20 detections of which are from the most recent incursion described here (Fig. 3). Overall, H5N5 viruses have been detected in eight species of birds and three species of mammals to date in Canada. Among birds, the viruses have been predominantly detected in marine species, specifically gulls and Northern Fulmars (*Fulmarus glacialis*). However, 9 out of the 61 cases in Canada were detected in American Crows that were found dead, and all 19 cases from Japan are also from corvids (Fig. 1). Given the scavenging habits of corvids (Forbes et al. 2022), it is not surprising that widespread detections have been made in these birds. These detections in scavenging birds such as corvids also indicate the virus is most likely more prevalent on the landscape in wild birds, but it is not being detected via live bird sampling programs. Future work should consider why these H5N5 viruses are being detected in dead birds and mammals, but not in otherwise apparently healthy live birds that form a large component of AIV surveillance taking place over this time in the region.

Outside of Canada, Norway is the only country that has reported mammalian cases of H5N5 as of June 2024 (World Organisation for Animal Health (WOAH) 2024). H5N5 has not been detected in poultry so far, in contrast to H5N1 that was detected in poultry on the Avalon Peninsula of Newfoundland and Labrador roughly a month after the initial detection in a wild bird (Caliendo et al. 2022). Limited spread of H5N5 or increased biosecurity measures for HPAIV may be contributing to this difference.

Phylogeographic analysis of H5N5 viruses depicts their global dissemination routes (Fig. 3). The earliest detection of a similar H5N5 virus was in 2020 in Russia (Rostov) in a swan (species not reported). In 2021, related viruses appeared elsewhere in Russia (Kalmykia, Astrakhan, and Dagestan), Romania, and Bulgaria (Fig. 1) (Zinyakov et al. 2022). By 2022, the virus spread to Norway, where it infected a range of bird species and was then transmitted into Atlantic Canada via migratory birds (Figs 2 and 3) (Erdelyan et al. 2024). Among the hosts infected in different regions during these movements were multiple species of gulls (Fig. 1). The H5N5 viruses continued to circulate in Europe followed by another transmission into Atlantic Canada. Norway appears to be a clear source of dissemination (Figs 1 and 3) (Bøe et al. 2024) as the virus was transmitted from there into the UK, Japan, Iceland, and Canada in 2023. Related viruses have subsequently been detected in the Netherlands, the UK, and Germany in 2024 (Fig. 1). Infections again occurred in multiple species of gulls during these movements, implicating them as particularly important in the circulation of these H5N5 viruses. When phylogeographic analysis was repeated with a reduced dataset to prevent bias, a similar pattern was observed (Supplementary Fig. S3). While down-sampling can affect inferred migration events and provincial transmission differs between phylogeographic reconstructions (Liu et al. 2022), at the country level, the dissemination pattern since H5N5 detection in Norway remains consistent.

### Notable features of the H5N5 sequences

The Rostov-like H5N5 viruses (Zinyakov et al. 2022) that have been detected since 2023 have a deletion in the neuraminidase (NA) gene that results in a 22 amino acid stalk truncation in the NA protein (H5N5-Δstalk) (Erdelyan et al. 2024) and have HA-A156S/T mutations (mature H5 numbering), allowing glycosylation of HA-N154 (Gao et al. 2018). Ancestral sequence reconstruction was performed and the codon corresponding to HA-156 was inferred to have undergone three separate convergent G514A/T events. These represent two HA-G514A mutations, which correspond to a HA-A156T substitution, and one HA-G514T mutation, corresponding to HA-A156S (Fig. S2). The new H5N5 viruses described here represent the third independent instance of a mutation allowing glycosylation within the H5N5-Δstalk viruses (Fig. 1), whereas a similar mutation has not been detected in any of the 2118 Canadian H5N1 virus sequences available on GISAID to date (Signore et al. 2024). Recent evidence (Nataraj et al. 2024) suggests reduced HA glycosylation appears to select for NA with long stalks in clade 2.3.4.4b viruses, increasing viral fitness, contrasted with earlier H5 clades containing HA that was more glycosylated and had NA with a shorter stalk. This aligns with the previous suggestion that HA-NA balance may be critical for H5N5-Δstalk viruses (Erdelyan et al. 2024).

The HA protein plays a crucial role in binding to host cell receptors and facilitates viral entry. Mutations at HA-156 have significant implications for viral fitness, receptor binding, and transmission dynamics (Gao et al. 2018, Du et al. 2020, Antigua et al. 2022). The HA-A156T mutation significantly enhances binding affinity for avian receptors, promoting efficient viral entry and replication in birds (Du et al. 2020). Therefore, repeated evolution of these mutations from HA-A156 suggests a benefit for increased HA glycosylation to balance the NA deletion within avian hosts, facilitating rapid spread within these populations, whereas H5N5-Δstalk viruses show weak affinity for mammalian–host receptors (Erdelyan et al. 2024). Even with a strong avian receptor preference, greatly increased pathogenicity in mammals is observed (Erdelyan et al. 2024) compared to previous H5N1 strains detected in North America (Kandeil et al. 2023). Experiments to test the effects of the HA-A156S/T substitutions in the specific genomic context of these H5N5 viruses are needed to confirm these possibilities.

The H5N5-Δstalk viruses circulating globally do not appear to have been part of any subsequently detected reassortment events. One H5N1 infection from Japan had N5-Δstalk reads in the genomic data indicating a potential coinfection, but no detection of a reassortant was subsequently described (Hew et al. 2024). This contrasts with previous reports of clade 2.3.4.4b H5N5 viruses readily undergoing reassortment events followed by rapid displacement of these reassortants by other H5Nx viruses (Pohlmann et al. 2018, King et al. 2022).

Samples from the most recent third H5N5 incursion reported here show the substitution PB2-E627K, a mammalian adaptation that increases virulence (Hatta et al. 2001), arose independently four separate times (Fig. 1). The substitution arose three times in mammals and was also detected in an American Crow (Fig. 1). Viruses from a raccoon, a skunk, and two gulls in Canada (Erdelyan et al. 2024), a cluster of viruses detected in Norway, England, Germany, and the Netherlands, and another group of four sequences from Japan have also been identified with this mutation (Fig. 1).

Two viruses from crows collected in Charlottetown, Prince Edward Island were found to contain PB2-D701N substitutions (Fig. 1). This substitution is associated with mammalian adaptation and pathogenicity (Li et al. 2005) and an increase polymerase activity and viral replication (Czudai-Matwich et al. 2014). This substitution has not previously been detected in Rostov-like strains (Zinyakov et al. 2022). As discussed earlier, crows are scavengers and they most likely acquired the virus from an infected mammal or bird, as previously suggested (Erdelyan et al. 2024, Hew et al. 2024). Interestingly, a virus from a skunk from Mount Stewart, Prince Edward Island that was the most similar to these two viruses did not have this mutation but contained PB2-E627K.

Out of the 20 new sequences, six were from viruses passaged in eggs and 14 were sequenced directly from the original samples (Supplementary Table S1). While egg-adaptative mutations can occur, A/Raccoon/NS/FAV-0079-1/2024 and A/Raccoon/PEI/FAV-0108-1/2024 are both from mammals and contain a PB2-E627K substitution, which is unlikely to occur in clade 2.3.4.4b viruses from avian species. The PB2-D701N and PB2-E627K mutations have synergistic effects on viral replication (Arai et al. 2018), but no H5N5 viruses with both mutations have been detected as of yet. The first H5N5 virus detected in Canada in 2023 lacks both PB2 substitutions and displays a strong binding preference for avian receptors, although it displays 100% mortality in ferrets (Erdelyan et al. 2024).

### Changes in global H5Nx circulation have facilitated repeated trans-Atlantic incursions

The frequent repeated introduction of H5Nx viruses into North America is a recent phenomenon and is possibly due to the critical shift of the epicentre of H5Nx HPAIV circulation. Although the initial outbreaks by these H5Nx viruses were largely restricted to Asia (Chen et al. 2005, 2006, Ip et al. 2016), since 2014 the outbreaks have been more widespread (Ip et al. 2015, Lee et al. 2018). While the viruses for the 2014–15 (The Global Consortium for H5N8 and Related Influenza Viruses 2016) and the 2016–17 (Lycett et al. 2020) epizootics had originated in China, the H5N8 viruses associated with the 2020–21 outbreaks were first reported in African poultry, spread to Eurasia, and then the H5N1 viruses that started the 2021–22 expansion into the Americas emerged from these H5N8 viruses (Xie et al. 2023). This highlights the regional evolution of these viruses and a shift of the emergence of clade 2.3.4.4b viruses from Asia to Africa (Xie et al. 2023). Moreover, due to the continuous circulation of H5Nx viruses in Europe, it has been suggested that the viruses have become enzootic there (Pohlmann et al. 2022). The shift from a more constrained seasonal pattern to a potentially continuous enzootic presence has important implications for HPAIV surveillance of domestic and wild birds in Europe and other parts of the world (Pohlmann et al. 2022). The clade 2.3.4.4b viruses we identified are reassortants that originated in Europe and then spread and diversified. In 2023, H5N5 was also detected in Japan, the first detection near the Pacific region and the virus was most similar to European sequences (Hew et al. 2024). While this H5N5 virus was first detected in Russia, it appears to radiate from Norway to other countries since 2022 (Fig. 3) (Bøe et al. 2024). The results presented here along with other studies collectively demonstrate that the clade 2.3.4.4b viruses are repeatedly and actively moving from Europe to North America across the Atlantic Ocean (Caliendo et al. 2022, Alkie et al. 2023a, Erdelyan et al. 2024). This suggests that IAV monitoring in Canada aimed at tracking regional and early detection patterns should focus on Atlantic Canada as these repeated incursions of the virus into this region suggest that wildlife in the North Atlantic contribute to a transmission conduit from Europe, and then onwards to the rest of North America. It should be noted that IAVs are also spreading from Europe to Asia and subsequently to North America via the Pacific Ocean (Alkie et al. 2022, Ramey et al. 2024), but this transmission route appears to be resulting in relatively less virus transmission into North American flyways at this time.

## Conclusions

This study describes the fifth incursion and dissemination of a Eurasian 2.3.4.4b clade H5Nx virus into Atlantic Canada since November 2021. These viruses have been found in a wide range of wild bird species and have been transmitted to mammals. Phylogenetic analyses indicated that this H5N5 strain was a novel introduction from Eurasia and was not the continued spread and dissemination of H5N5 viruses that were detected earlier in 2023. Several amino acid changes detected in the HA, NA, and PB2 sequences of these H5N5 viruses are known to affect pathogenicity and transmission, HA glycosylation, and the ability to bind to avian receptors. The presence of these mutations suggests ongoing selective pressure for adaptation to avian hosts, which may facilitate the rapid spread among wild birds and result in additional spillovers to mammals. Our findings provide direct evidence that these H5N5 viruses can infect and be transmitted by a broad range of host species, and as such have quickly become globally disseminated. Continued surveillance of IAVs in wild animals with a focus in high-transmission zones, such as Atlantic Canada is crucial to track the evolution of these viruses during their ongoing circulation and to assess potential impacts they may have on wildlife and public health.

## Supplementary Material

veae111_Supp

## Data Availability

The 20 genomic sequences have been deposited on GISAID under Isolate IDs EPI_ISL_19260099 to EPI_ISL_19260118. Raw sequence data were submitted to the NCBI Short Read Archive (BioProject ID PRJNA1182792).

## References

[R1] Alkie TN , Byrne AMP, Jones MEB et al. Recurring trans-Atlantic incursion of clade 2.3.4.4b H5N1 viruses by long distance migratory birds from northern Europe to Canada in 2022/2023. *Viruses*2023a;15:1836. doi: 10.3390/v15091836 PMC10536465 37766243

[R2] Alkie TN , Cox S, Embury-Hyatt C et al. Characterization of neurotropic HPAI H5N1 viruses with novel genome constellations and mammalian adaptive mutations in free-living mesocarnivores in Canada. *Emerg Microbes Infect*2023b;12:2186608. doi: 10.1080/22221751.2023.2186608 PMC10026807 36880345

[R3] Alkie TN , Lopes S, Hisanaga T et al. A threat from both sides: multiple introductions of genetically distinct H5 HPAI viruses into Canada via both East Asia-Australasia/Pacific and Atlantic flyways. *Virus Evol* 2022;8:veac077. doi: 10.1093/ve/veac077 PMC9463990 36105667

[R4] Antigua KJC , Baek YH, Choi W-S et al. Multiple HA substitutions in highly pathogenic avian influenza H5Nx viruses contributed to the change in the NA subtype preference. *Virulence* 2022;13:990–1004. doi: 10.1080/21505594.2022.2082672 36560870 PMC9176248

[R5] Arai Y , Kawashita N, Hotta K et al. Multiple polymerase gene mutations for human adaptation occurring in Asian H5N1 influenza virus clinical isolates. *Sci Rep* 2018;8:13066. doi: 10.1038/s41598-018-31397-3 PMC6117316 30166556

[R6] Avery-Gomm S , Barychka T, English M et al. Wild bird mass mortalities in eastern Canada associated with the Highly Pathogenic Avian Influenza A (H5N1) virus, 2022. *Ecosphere* 2024;15:e4980.doi: 10.1002/ecs2.4980

[R7] Bielejec F , Baele G, Vrancken B et al. SpreaD3: interactive visualization of spatiotemporal history and trait evolutionary processes. *Mol Biol Evol* 2016;33:2167–69. doi: 10.1093/molbev/msw082 27189542 PMC6398721

[R8] Bøe CA , Fiskebeck EMLZ, Reiten MR et al. Emergence of highly pathogenic avian influenza viruses H5N1 and H5N5 in white-tailed eagles, 2021–2023. *J Gen Virol* 2024;105:002035. doi: 10.1099/jgv.0.002035 PMC11529892 39485726

[R9] Caliendo V , Lewis NS, Pohlmann A et al. Transatlantic spread of highly pathogenic avian influenza H5N1 by wild birds from Europe to North America in 2021. *Sci Rep* 2022;12:11729. doi: 10.1038/s41598-022-13447-z PMC9276711 35821511

[R10] Chen H , Smith GJD, Li KS et al. Establishment of multiple sublineages of H5N1 influenza virus in Asia: implications for pandemic control. *Proc Natl Acad Sci U S A* 2006;103:2845–50. doi: 10.1073/pnas.0511120103 16473931 PMC1413830

[R11] Chen H , Smith GJD, Zhang SY et al. Avian flu H5N1 virus outbreak in migratory waterfowl. *Nature* 2005;436:191–92. doi: 10.1038/nature03974 16007072

[R12] Chestakova IV , van der Linden A, Bellido Martin B et al. High number of HPAI H5 virus infections and antibodies in wild carnivores in the Netherlands, 2020–2022. *Emerg Microbes Infect* 2023;12:2270068. doi: 10.1080/22221751.2023.2270068 PMC10732216 37842795

[R13] Czudai-Matwich V , Otte A, Matrosovich M et al. PB2 mutations D701N and S714R promote adaptation of an influenza H5N1 virus to a mammalian host. *J Virol* 2014;88:8735–42. doi: 10.1128/JVI.00422-14 24899203 PMC4136279

[R14] de Bruin ACM , Funk M, Spronken MI et al. Hemagglutinin subtype specificity and mechanisms of highly pathogenic avian influenza virus genesis. *Viruses* 2022;14:1566. doi: 10.3390/v14071566 PMC9321182 35891546

[R15] Du W , Wolfert MA, Peeters B et al. Mutation of the second sialic acid-binding site of influenza A virus neuraminidase drives compensatory mutations in hemagglutinin. *PLOS Pathog* 2020;16:e1008816. doi: 10.1371/journal.ppat.1008816 PMC7480853 32853241

[R16] Erdelyan CNG , Kandeil A, Signore AV et al. Multiple transatlantic incursions of highly pathogenic avian influenza clade 2.3.4.4b A(H5N5) virus into North America and spillover to mammals. *Cell Rep* 2024;43:114479. doi: 10.1016/j.celrep.2024.114479 PMC11305400 39003741

[R17] Fereidouni S , Starick E, Karamendin K et al. Genetic characterization of a new candidate hemagglutinin subtype of influenza A viruses. *Emerg Microbes Infect* 2023;12:2225645. doi: 10.1080/22221751.2023.2225645 PMC10308872 37335000

[R18] Forbes SL , Samson C, Watson CJ. Seasonal impact of scavenger guilds as taphonomic agents in central and northern Ontario, Canada. *J Forensic Sci* 2022;67:2203–17. doi: 10.1111/1556-4029.15122 35957551

[R19] Gao R , Gu M, Liu K et al. T160A mutation-induced deglycosylation at site 158 in hemagglutinin is a critical determinant of the dual receptor binding properties of clade 2.3.4.4 H5NX subtype avian influenza viruses. *Vet Microbiol* 2018;217:158–66. doi: 10.1016/j.vetmic.2018.03.018 29615249

[R20] Giacinti JA , Signore AV, Jones MEB et al. Avian influenza viruses in wild birds in Canada following incursions of highly pathogenic H5N1 virus from Eurasia in 2021–2022. *mBio* 2024;15:e03203–23. doi: 10.1128/mbio.03203-23 39012149 PMC11323545

[R21] The Global Consortium for H5N8 and Related Influenza Viruses . Role for migratory wild birds in the global spread of avian influenza H5N8. *Science* 2016;354:213–17. doi: 10.1126/science.aaf8852 27738169 PMC5972003

[R22] Guo Y , Xu X, Wan X. Genetic characterization of an avian influenza A (H5N1) virus isolated from a sick goose in China. *Chin J Exp Clin Virol* 1998;12:322–25. 12526344

[R23] Hatta M , Gao P, Halfmann P et al. Molecular basis for high virulence of Hong Kong H5N1 influenza A viruses. *Science* 2001;293:1840–42. doi: 10.1126/science.1062882 11546875

[R24] Hew YL , Hiono T, Monne I et al. Cocirculation of genetically distinct highly pathogenic avian influenza H5N5 and H5N1 viruses in crows, Hokkaido, Japan. *Emerg Infect Dis* 2024;30:1912. doi: 10.3201/eid3009.240356 PMC11346982 39106453

[R25] Hicks JT , Dimitrov KM, Afonso CL et al. Global phylodynamic analysis of avian paramyxovirus-1 provides evidence of inter-host transmission and intercontinental spatial diffusion. *BMC Evol Biol* 2019;19:108. doi: 10.1186/s12862-019-1431-2 PMC6534909 31126244

[R26] Ip HS , Dusek RJ, Bodenstein B et al. High rates of detection of clade 2.3.4.4 highly pathogenic avian influenza H5 viruses in wild birds in the Pacific Northwest during the winter of 2014–15. *Avian Dis* 2016;60:354–58. doi: 10.1637/11137-050815-Reg 27309079

[R27] Ip HS , Torchetti MK, Crespo R et al. Novel Eurasian highly pathogenic avian influenza A H5 viruses in wild birds, Washington, USA, 2014. *Emerg Infect Dis* 2015;21:886–90. doi: 10.3201/eid2105.142020 25898265 PMC4412248

[R28] Kalyaanamoorthy S , Minh BQ, Wong TKF et al. ModelFinder: fast model selection for accurate phylogenetic estimates. *Nat Methods* 2017;14:587–89. doi: 10.1038/nmeth.4285 28481363 PMC5453245

[R29] Kandeil A , Patton C, Jones JC et al. Rapid evolution of A(H5N1) influenza viruses after intercontinental spread to North America. *Nat Commun* 2023;14:3082. doi: 10.1038/s41467-023-38415-7 PMC10227026 37248261

[R30] King J , Harder T, Globig A et al. Highly pathogenic avian influenza virus incursions of subtype H5N8, H5N5, H5N1, H5N4, and H5N3 in Germany during 2020-21. *Virus Evol* 2022;8:veac035. doi: 10.1093/ve/veac035 PMC9037367 35478715

[R31] Kruczkiewicz P , Nguyen N, Lung O. CFIA-NCFAD/nf-flu. 2024.

[R32] Lair S , Quesnel L, Signore AV et al. Outbreak of highly pathogenic avian influenza A(H5N1) virus in seals, St. Lawrence Estuary, Quebec, Canada. *Emerg Infect Dis* 2024;30:1133–43. doi: 10.3201/eid3006.231033 38781927 PMC11138997

[R33] Lane JV , Jeglinski JWE, Avery-Gomm S et al. High pathogenicity avian influenza (H5N1) in Northern Gannets (*Morus bassanus*): Global spread, clinical signs and demographic consequences. *Ibis* 2024;166:633–50. doi: 10.1111/ibi.13275

[R34] Lee D-H , Torchetti MK, Hicks J et al. Transmission dynamics of highly pathogenic avian influenza virus A(H5Nx) clade 2.3.4.4, North America, 2014–2015. *Emerg Infect Dis* 2018;24:1840–48. doi: 10.3201/eid2410.171891 30226167 PMC6154162

[R35] Leguia M , Garcia-Glaessner A, Muñoz-Saavedra B et al. Highly pathogenic avian influenza A (H5N1) in marine mammals and seabirds in Peru. *Nat Commun* 2023;14:5489. doi: 10.1038/s41467-023-41182-0 PMC10484921 37679333

[R36] Lemey P , Rambaut A, Drummond AJ et al. Bayesian phylogeography finds its roots. *PLoS Comput Biol* 2009;5:e1000520. doi: 10.1371/journal.pcbi.1000520 PMC2740835 19779555

[R37] Lewis NS , Banyard AC, Whittard E et al. Emergence and spread of novel H5N8, H5N5 and H5N1 clade 2.3.4.4 highly pathogenic avian influenza in 2020. *Emerg Microbes Infect* 2021;10:148–51. doi: 10.1080/22221751.2021.1872355 33400615 PMC7832535

[R38] Li H , Birol I. Minimap2: pairwise alignment for nucleotide sequences. *Bioinformatics* 2018;34:3094–100. doi: 10.1093/bioinformatics/bty191 29750242 PMC6137996

[R39] Li Z , Chen H, Jiao P et al. Molecular basis of replication of duck H5N1 influenza viruses in a mammalian mouse model. *J Virol* 2005;79:12058–64. doi: 10.1128/JVI.79.18.12058-12064.2005 16140781 PMC1212590

[R40] Liu P , Song Y, Colijn C et al. The impact of sampling bias on viral phylogeographic reconstruction. Falcão De Oliveira E (ed.). *PLOS Glob Public Health* 2022;2:e0000577. doi: 10.1371/journal.pgph.0000577 PMC10021582 36962555

[R41] Lycett SJ , Pohlmann A, Staubach C et al. Genesis and spread of multiple reassortants during the 2016/2017 H5 avian influenza epidemic in Eurasia. *Proc Natl Acad Sci U S A* 2020;117:20814–25. doi: 10.1073/pnas.2001813117 32769208 PMC7456104

[R42] Martin DP , Varsani A, Roumagnac P et al. RDP5: a computer program for analyzing recombination in, and removing signals of recombination from, nucleotide sequence datasets. *Virus Evol* 2021;7:veaa087. doi: 10.1093/ve/veaa087 PMC8062008 33936774

[R43] Nataraj R , Chandra A, Kesavardhana S. Avian influenza virus neuraminidase stalk length and haemagglutinin glycosylation patterns reveal molecularly directed reassortment promoting the emergence of highly pathogenic clade 2.3.4.4b A (H5N1) viruses. 2024.

[R44] Nguyen L-T , Schmidt HA, Von Haeseler A et al. IQ-TREE: a fast and effective stochastic algorithm for estimating maximum-likelihood phylogenies. *Mol Biol Evol* 2015;32:268–74. doi: 10.1093/molbev/msu300 25371430 PMC4271533

[R45] Olsen B , Munster VJ, Wallensten A et al. Global patterns of influenza A virus in wild birds. *Science* 2006;312:384–88. doi: 10.1126/science.1122438 16627734

[R46] Plaza PI , Gamarra-Toledo V, Rodríguez Euguí J et al. Pacific and Atlantic sea lion mortality caused by highly pathogenic Avian Influenza A(H5N1) in South America. *Travel Med Infectious Dis* 2024;59:102712. doi: 10.1016/j.tmaid.2024.102712 38461878

[R47] Pohlmann A , King J, Fusaro A et al. Has epizootic become enzootic? Evidence for a fundamental change in the infection dynamics of highly pathogenic avian influenza in Europe, 2021. *mBio* 2022;13:e00609–22. doi: 10.1128/mbio.00609-22 35726917 PMC9426456

[R48] Pohlmann A , Starick E, Grund C et al. Swarm incursions of reassortants of highly pathogenic avian influenza virus strains H5N8 and H5N5, clade 2.3.4.4b, Germany, winter 2016/17. *Sci Rep* 2018;8:15. doi: 10.1038/s41598-017-16936-8 PMC5758748 29311555

[R49] Ramey AM , Hill NJ, DeLiberto TJ et al. Highly pathogenic avian influenza is an emerging disease threat to wild birds in North America. *J Wildl Manage* 2022;86:e22171. doi: 10.1002/jwmg.22171

[R50] Ramey AM , Scott LC, Ahlstrom CA et al. Molecular detection and characterization of highly pathogenic H5N1 clade 2.3.4.4b avian influenza viruses among hunter-harvested wild birds provides evidence for three independent introductions into Alaska. *Virology* 2024;589:109938. doi: 10.1016/j.virol.2023.109938 37977084

[R51] Ruiz-Saenz J , Martinez-Gutierrez M, Pujol FH. Multiple introductions of highly pathogenic avian influenza H5N1 clade 2.3.4.4b into South America. *Travel Med Infectious Dis* 2023;53:102591. doi: 10.1016/j.tmaid.2023.102591 37201592

[R52] Sagulenko P , Puller V, Neher RA. TreeTime: maximum-likelihood phylodynamic analysis. *Virus Evol* 2018;4:vex042. doi: 10.1093/ve/vex042 PMC5758920 29340210

[R53] Shu Y , McCauley J. GISAID: Global initiative on sharing all influenza data – from vision to reality. *Eurosurveillance* 2017;22:30494. doi: 10.2807/1560-7917.ES.2017.22.13.30494 PMC5388101 28382917

[R54] Signore A , Giacinti J, Jones MEB et al. Successive lineage replacement by descendant reassortants in the North American A(H5N1) outbreak suggests compounding fitness increases. 2024. 10.1126/sciadv.adu4909 PMC12239939 40632842

[R55] Smith GJD , Donis RO. World Health Organization/World Organisation for Animal Health/Food and Agriculture Organization (WHO/OIE/FAO) H5 Evolution Working Group. Nomenclature updates resulting from the evolution of avian influenza A(H5) virus clades 2.1.3.2a, 2.2.1, and 2.3.4 during 2013–2014. *Influenza Other Respir Viruses* 2015;9:271–76. doi: 10.1111/irv.12324 25966311 PMC4548997

[R56] Spackman E , Senne DA, Myers TJ et al. Development of a real-time reverse transcriptase PCR assay for type A influenza virus and the avian H5 and H7 hemagglutinin subtypes. *J Clin Microbiol* 2002;40:3256–60. doi: 10.1128/JCM.40.9.3256-3260.2002 12202562 PMC130722

[R57] Stech J , Mettenleiter TC. Virulence determinants of high-pathogenic avian influenza viruses in gallinaceous poultry. *Future Virol* 2013;8:459–68. doi: 10.2217/fvl.13.27

[R58] Suchard MA , Lemey P, Baele G et al. Bayesian phylogenetic and phylodynamic data integration using BEAST 1.10. *Virus Evol* 2018;4:vey016. doi: 10.1093/ve/vey016 PMC6007674 29942656

[R59] Swayne DE . Understanding the complex pathobiology of high pathogenicity avian influenza viruses in birds. *Avian Dis* 2007;51:242–49. doi: 10.1637/7763-110706-REGR.1 17494560

[R60] Swayne DE , Suarez DL. Highly pathogenic avian influenza. *Revue Scientifique Et Technique OIE* 2000;19:463–82. doi: 10.20506/rst.19.2.1230 10935274

[R61] Tavaré S . Some probabilistic and statistical problems in the analysis of DNA sequences. *Lectures on Mathematics in the Life Sciences* 1986;17:57–86.

[R62] Terregino C Capua I . Conventional diagnosis of avian influenza. In: Capua I, Alexander DJ (eds), *Avian Influenza and Newcastle Disease*. Milano: Springer Milan, 2009, 73–85.

[R63] Ulloa M , Fernández A, Ariyama N et al. Mass mortality event in South American sea lions (*Otaria flavescens*) correlated to highly pathogenic avian influenza (HPAI) H5N1 outbreak in Chile. *Vet Q* 2023;43:1–10. doi: 10.1080/01652176.2023.2265173 PMC10588531 37768676

[R64] Weingartl HM , Berhane Y, Hisanaga T et al. Genetic and pathobiologic characterization of pandemic H1N1 2009 influenza viruses from a naturally infected swine herd. *J Virol* 2010;84:2245–56. doi: 10.1128/JVI.02118-09 20015998 PMC2820904

[R65] Wight J , Rahman I, Wallace HL et al. Avian influenza virus circulation and immunity in a wild urban duck population prior to and during a highly pathogenic H5N1 outbreak. *Vet Res* 2024;55:154. doi: 10.1186/s13567-024-01397-5 PMC11585116 39578905

[R66] World Organisation for Animal Health (WOAH) . Norway - Influenza A Viruses of High Pathogenicity (Inf. with) (Non-Poultry Including Wild Birds) (2017-) - Follow up Report 12. The World Animal Health Information System, 2024.

[R67] Wu Y , Wu Y, Tefsen B et al. Bat-derived influenza-like viruses H17N10 and H18N11. *Trends Microbiol* 2014;22:183–91. doi: 10.1016/j.tim.2014.01.010 24582528 PMC7127364

[R68] Xie R , Edwards KM, Wille M et al. The episodic resurgence of highly pathogenic avian influenza H5 virus. *Nature* 2023;622:810–17. doi: 10.1038/s41586-023-06631-2 37853121

[R69] Youk S , Torchetti MK, Lantz K et al. H5N1 highly pathogenic avian influenza clade 2.3.4.4b in wild and domestic birds: introductions into the United States and reassortments, December 2021–April 2022. *Virology* 2023;587:109860. doi: 10.1016/j.virol.2023.109860 37572517

[R70] Yu G , Smith DK, Zhu H et al. ggtree: an r package for visualization and annotation of phylogenetic trees with their covariates and other associated data. *Meth Ecol Evolut* 2017;8:28–36. doi: 10.1111/2041-210X.12628

[R71] Zhou B , Donnelly ME, Scholes DT et al. Single-reaction genomic amplification accelerates sequencing and vaccine production for classical and swine origin human influenza A viruses. *J Virol* 2009;83:10309–13. doi: 10.1128/JVI.01109-09 19605485 PMC2748056

[R72] Zinyakov N , Andriyasov A, Zhestkov P et al. Analysis of avian influenza (H5N5) viruses isolated in the southwestern European part of the Russian Federation in 2020–2021. *Viruses* 2022;14:2725. doi: 10.3390/v14122725 PMC9783257 36560728

