# Supplementary material for: Recurring incursions and dissemination of novel Eurasian-origin H5Nx avian influenza viruses in Atlantic Canada
Source: Virus Evol. 2024 Dec 17;10(1):veae111. doi: 10.1093/ve/veae111 (PMC11669315; doi:10.1093/ve/veae111)
Supplement: veae111_Supp [file veae111_supp.zip › suppl_data/RahmanErdelyan supplemental material_final.pdf]

Recurring incursions and dissemination of novel Eurasian-origin H5Nx avian influenza viruses in Atlantic Canada

Supplemental material

Table S1. Sources of sequenced Canadian H5N5 samples.

Sample	Material sequenced
WIN-AH-2023-FAV-0375-3	1 chicken egg passage, 2dpi
WIN-AH-2023-FAV-0405-1	Original sample
WIN-AH-2023-FAV-0405-2	Original sample
WIN-AH-2023-FAV-0405-3	Original sample
WIN-AH-2023-FAV-0430-7	Original sample
WIN-AH-2023-FAV-0430-9	1 chicken egg passage, 2dpi
WIN-AH-2023-FAV-0430-10	1 chicken egg passage, 4dpi
WIN-AH-2023-FAV-0430-11	Original sample
WIN-AH-2023-FAV-0430-12	1 chicken egg passage, 4dpi
WIN-AH-2023-FAV-0430-13	Original sample
WIN-AH-2023-FAV-0430-14	Original sample
WIN-AH-2024-FAV-0074-2	Original sample
WIN-AH-2024-FAV-0074-3	Original sample
WIN-AH-2024-FAV-0079-1	1 chicken egg passage, 2dpi
WIN-AH-2024-FAV-0093-1	Original sample
WIN-AH-2024-FAV-0108-1	1 chicken egg passage, 3dpi
WIN-AH-2024-FAV-0109-1	Original sample
WIN-AH-2024-FAV-0111-1	Original sample
WIN-AH-2024-FAV-0130-1	Original sample
WIN-AH-2024-FAV-0132-1	Original sample

Table S2. Sequences from GISAID used in this study (Excel file).

Recurring incursions and dissemination of novel Eurasian-origin H5Nx avian influenza viruses in Atlantic Canada

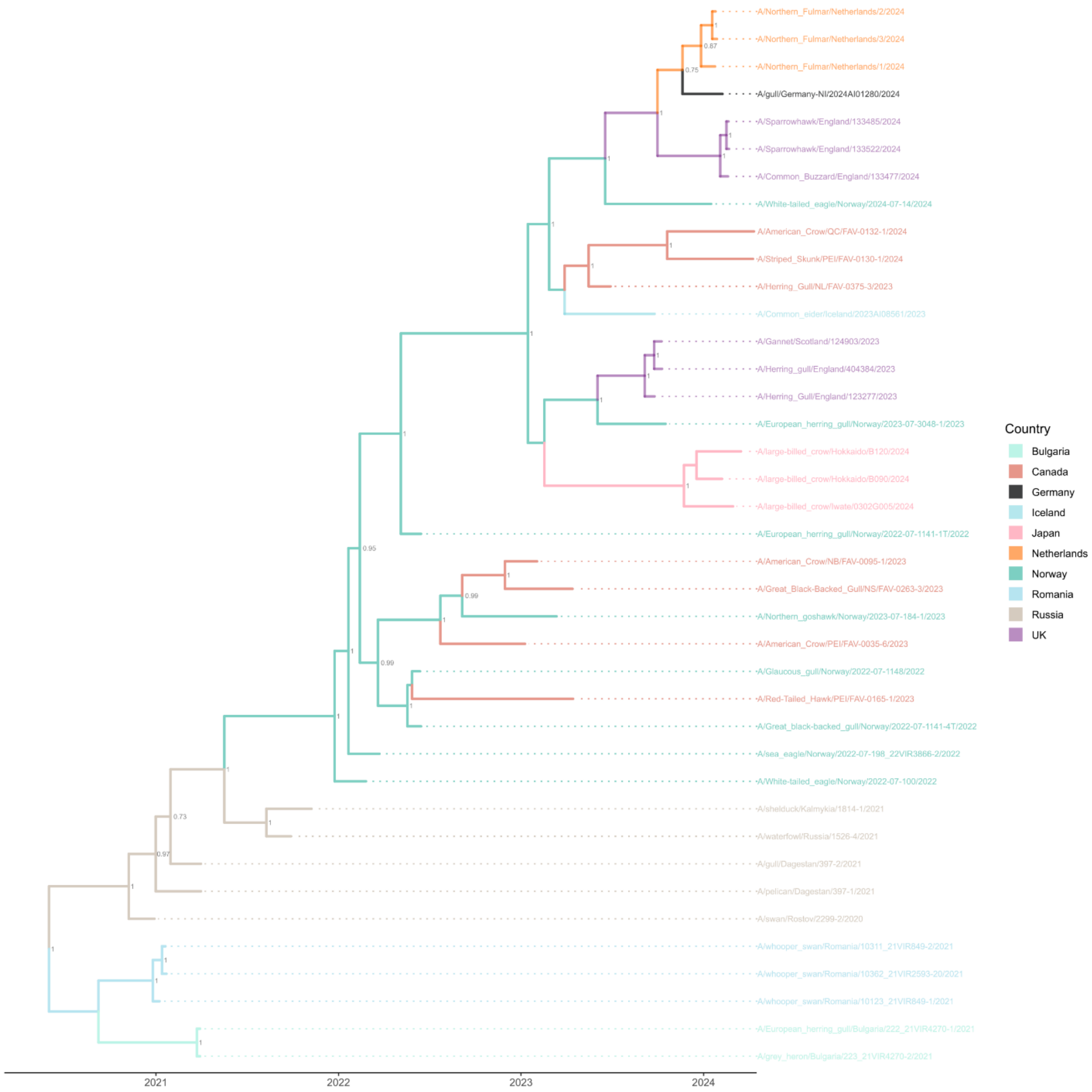

Figure S1. Bayesian time-resolved maximum clade credibility tree of a reduced H5N5 whole genome sequence dataset for viruses similar to the first detection, A/swan/Rostov/2299-2/2020 (H5N5), with a maximum of three sequences per region per cluster.

Recurring incursions and dissemination of novel Eurasian-origin H5Nx avian influenza viruses in Atlantic Canada

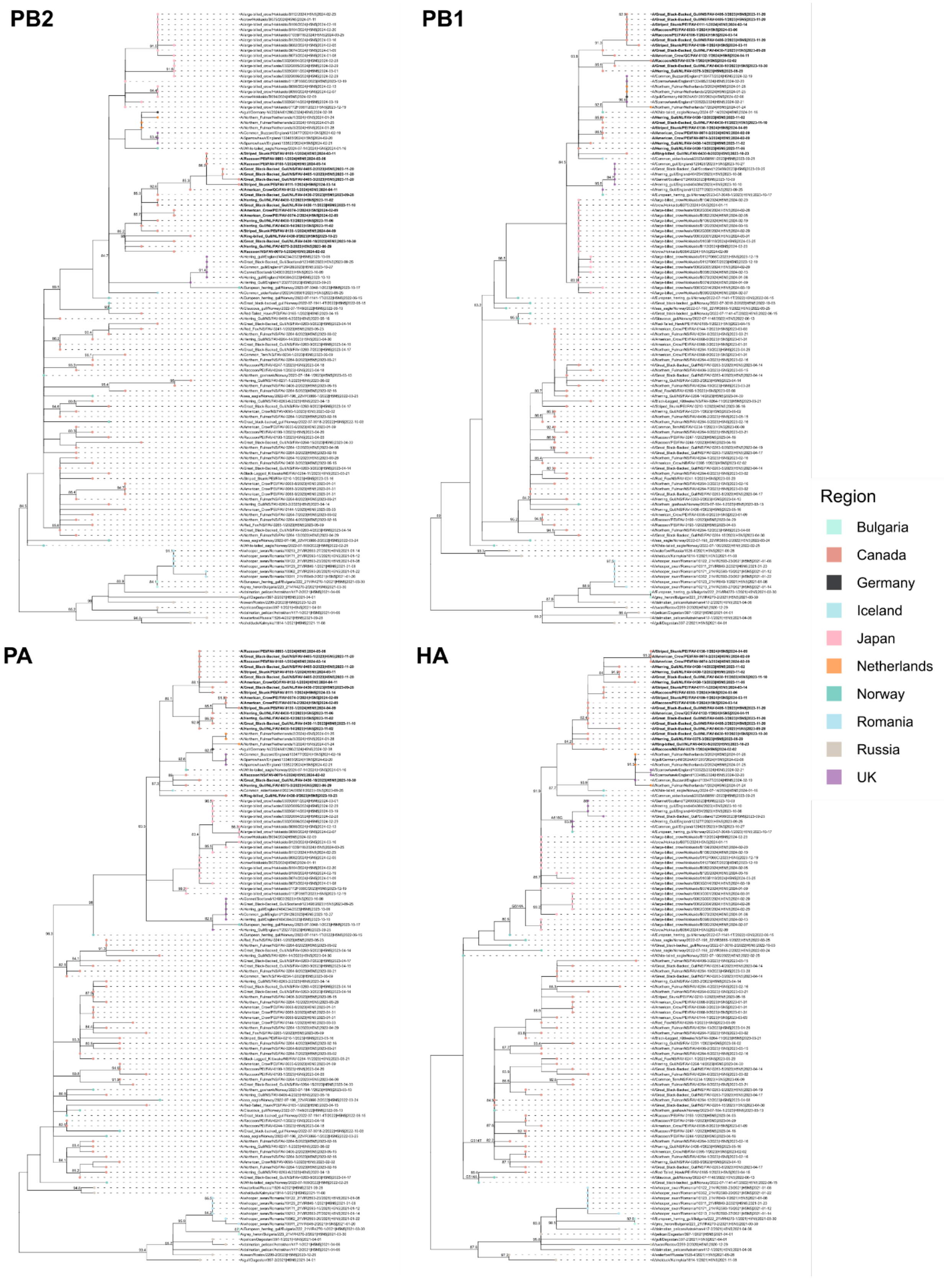

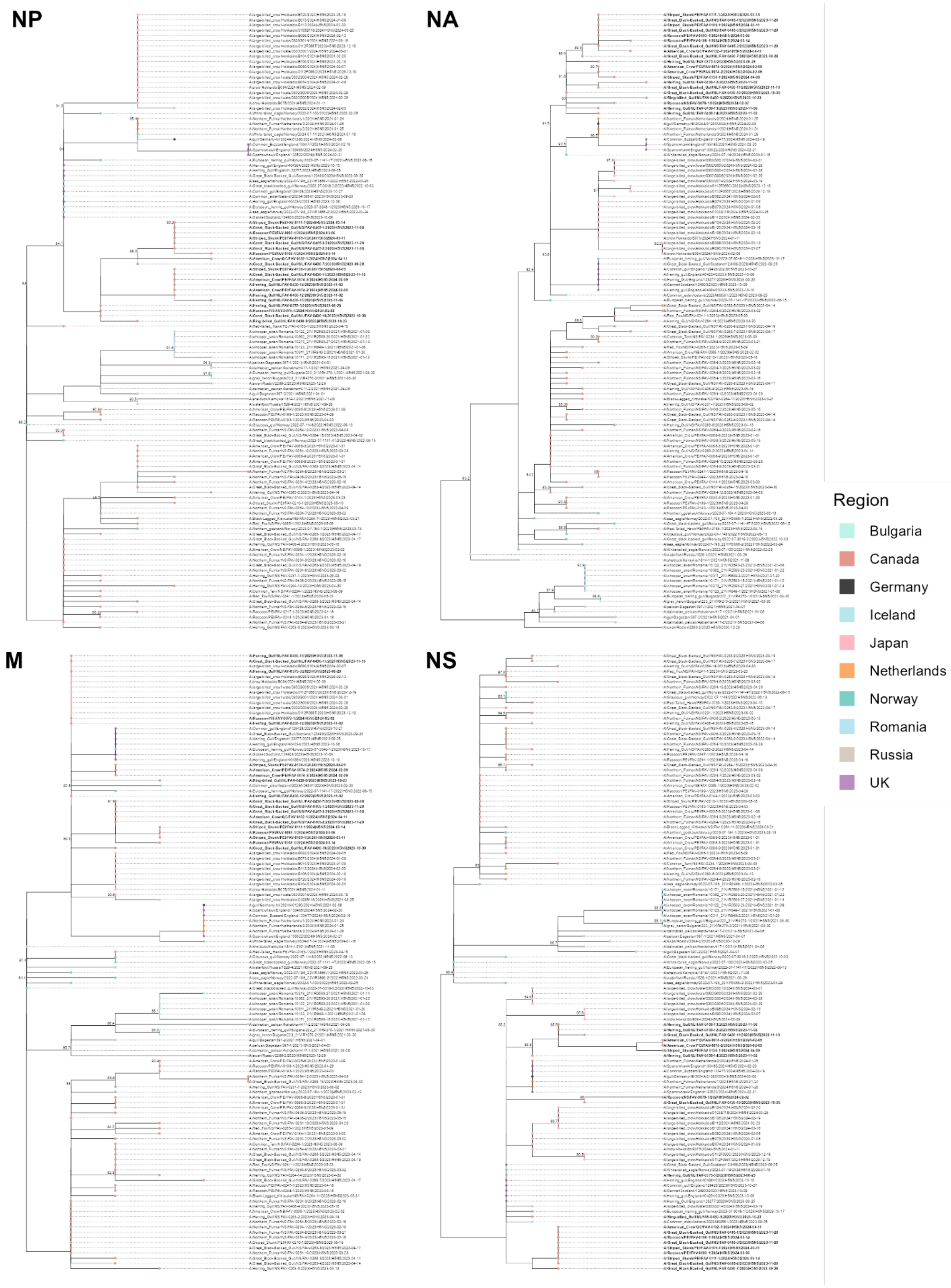
NP
NA
M
NS
Region
Bulgaria
Canada
Germany
Iceland
Japan
Netherlands
Norway
Romania
Russia
UK

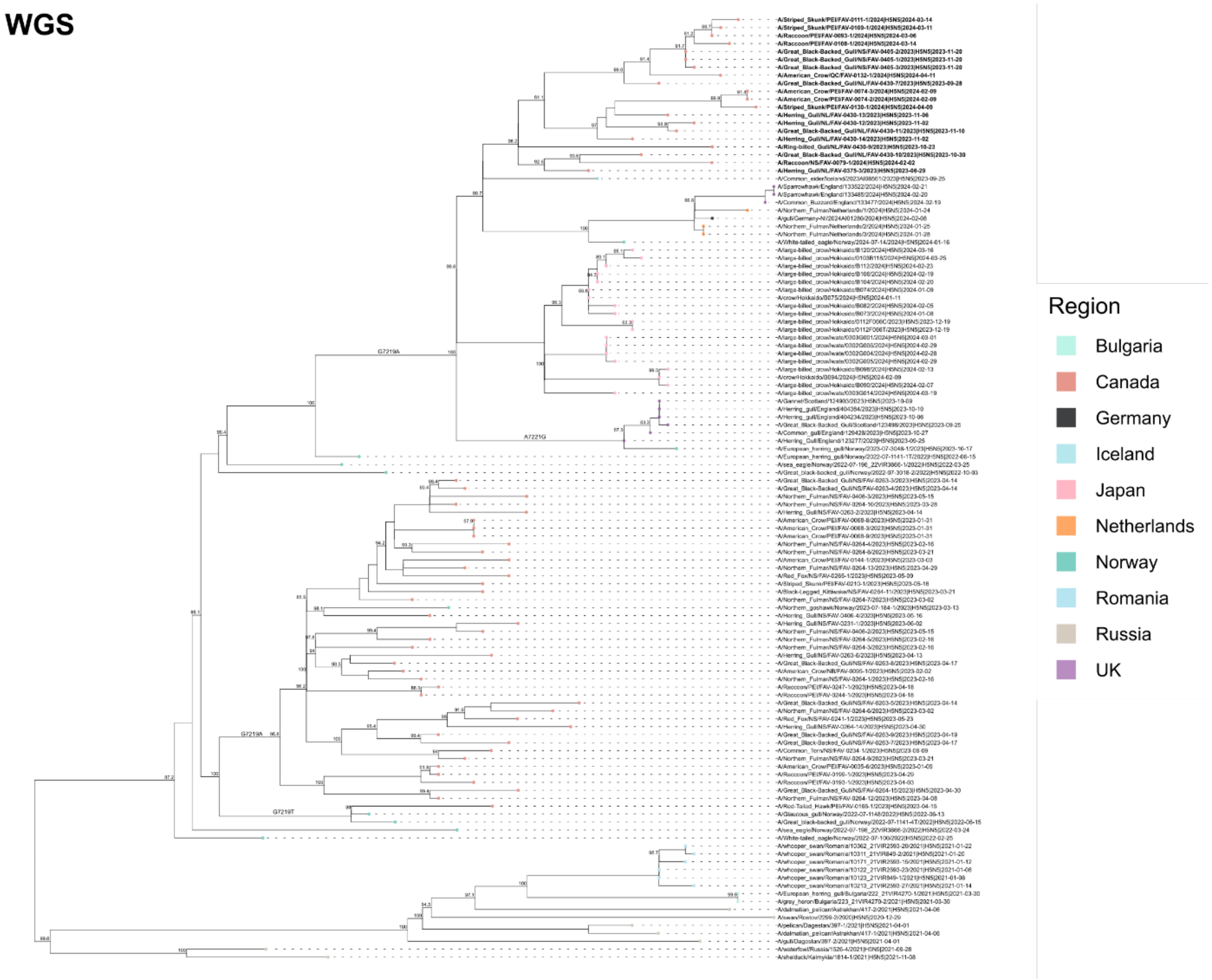

Figure S2. Maximum-likelihood phylogenetic trees of the segments and WGS from H5N5 viruses. HA and WGS trees show mutations from ancestral sequence reconstructions on the branches pertaining to the codon for HA-156 (mature H5 numbering) inferred by TreeTime. Values are from 1000 replicates of the Shimodaira–Hasegawa approximate likelihood ratio test.

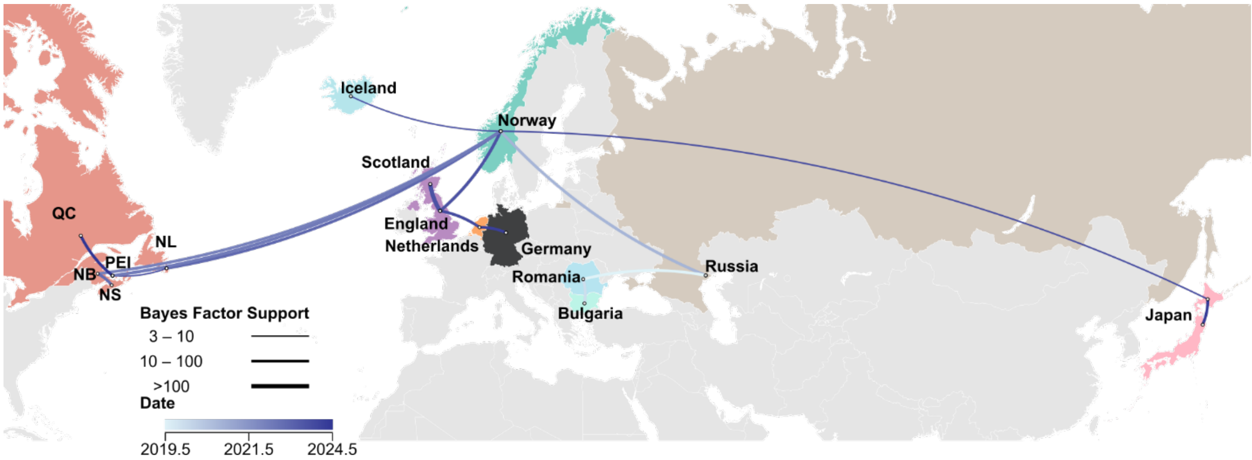

Figure S3. Phylogeographic reconstruction of H5N5 dispersal since the first detection in Russia in 2020 using a reduced H5N5 whole genome sequence dataset.
